# Difficulties in the Modeling of *E. coli* Spreading from Various Sources in a Coastal Marine Area

**DOI:** 10.3390/molecules27144353

**Published:** 2022-07-07

**Authors:** Lidia Wolska, Marek Kowalewski, Marta Potrykus, Vladyslav Redko, Bartosz Rybak

**Affiliations:** 1 Department of Environmental Toxicology, Faculty of Health Sciences, Medical University of Gdańsk, Dębowa Str. 23A, 80-204 Gdańsk, Poland; lidia.wolska@gumed.edu.pl (L.W.); marta.potrykus@gumed.edu.pl (M.P.); vladyslav.redko@gumed.edu.pl (V.R.); 2 Institute of Oceanography, University of Gdańsk, Av. Marszałka Piłsudskiego 46, 81-378 Gdynia, Poland; marek.kowalewski@ug.edu.pl

**Keywords:** mathematical model, *Escherichia coli* spread, microbial bathing water quality

## Abstract

Coastal and transitional waters are often used as bathing waters. In many regions, such activities play an important economic role. According to the European Union Bathing Water Directive (2006/7/EC) (BWD) the concentration of *Escherichia coli* in bathing water exceeding 500 CFU·100 mL^−1^ poses a high risk for bathers’ health. In order to safeguard public health, microbiological environmental monitoring is carried out, which has recently been supported or replaced by mathematical models detailing the spread of sanitary contamination. This study focuses on the problems and limitations that can be encountered in the process of constructing a mathematical model describing the spread of biological contamination by *E. coli* bacteria in coastal seawater. This and other studies point to the following problems occurring during the process of building and validating a model: the lack of data on loads of sanitary contamination (often connected with multiple sources of biological contamination inflow) makes the model more complex; *E. coli* concentrations higher than 250 CFU·100 mL^−1^ (low hazard for health) are observed very rarely, and are associated with great uncertainty; the impossibility of predicting the time and intensity of precipitation as well as stronger winds and rougher sea, which may be a significant source of *E. coli*. However, there is universal agreement that such models will be useful in managing bathing water quality and protecting public health, especially during big failures of the wastewater network.

## 1. Introduction

The process of environmental pollution, which has been occurring for hundreds of years, has clearly accelerated in the last century, and it has taken on dimensions unprecedented in the history of mankind, both in terms of quantity and variety of physical, chemical, and microbiological pollutants emitted into the environment [1]. This has resulted in the degradation of the environment, especially the aquatic one and has increased human health problems leading to chronic diseases or even premature deaths [2].

For over 50 years, the predominant focus has been on the use of specific technical solutions for discharging treated wastewater from wastewater treatment plants (WWTPs) into the waterways as an advantageous solution to effectively reduce the number of microorganisms present in the wastewater, which for various reasons (including WWTPs function failures) are not eliminated in the treatment process [3]. In general, treated wastewater is discharged several kilometers into the sea, where it is mixed with seawater through diffusers placed on the seabed. However, in this process, freshwater is pushed to the surface of the sea, forming so-called lenses. Could this strategy pose a risk to bathers and beach-goers in the vicinity of diffusers discharging wastewater into the sea? Factors such as hydrological conditions or wind direction can cause freshwater lenses to return towards land (beaches), transporting microorganisms, including pathogens that may worsen bathing water quality. Moreover, in the case of WWTP failure, significant quantities of contaminants and microorganisms, including fecal pathogens such as Enteropathogenic *Escherichia coli* and *Salmonella* spp., may be introduced into the sea with the wastewater discharge. Is there, therefore, a risk that people bathing in the sea could be infected with these bacteria? One of the health problems that may occur due to the increased fecal pathogen presence in the seawater is urinary tract infection (UTI). In 70–95% of UTI cases, the main cause of this illness is *E. coli* infection, less frequently a fungal or viral contagion. Although UTI is associated with a lack of personal hygiene and translocation of the fecal flora from the intestine to the bladder or the urethra, where its epithelium becomes colonized and infected [4], water reservoirs are also an important source of UTI-causing bacterial strains [5]. Therefore, sanitary services provide microbiological control of bathing sites, so people are generally aware of the presence of UTI-causing bacteria in the water. Nevertheless, these actions are limited in time and localized [6,7]. There are several groups of people that should monitor sanitary service announcements due to their increased risk of UTI, i.e., infants and children, elderly people, pregnant women, diabetics, patients suffering from multiple sclerosis or spinal cord injuries as well as individuals with acquired immunodeficiency syndrome or urological diseases [5,8,9,10,11]. Both UTI and urosepsis are treatable conditions, but delayed management can lead to severe consequences, such as kidney failure, septic shock, and death [12]. Drug-resistant *E. coli* are also detected in bathing waters [13]. Infection with such bacteria during sea bathing raises additional problems related to the treatment process.

Water bodies, including discharging areas, are important feeding and spawning zones for marine, anadromous, and freshwater ichthyofauna species. The abundance of individual species is variable and dependent on weather conditions. Unfortunately, the consumption of fish caught from the treated wastewater discharge areas can be considered an additional threat since enteropathogenic *E. coli* strains have been isolated from both farmed and free-living fish [14]. These microorganisms infect fish through their digestive tract, leading to inflammation of the gastric and intestinal mucosa. An increased concentration of *E. coli* in anthropopressed waters from treated wastewater can result in a rise in the number of infections in free-living fish, which can cause an epidemiological threat to humans who consume them. Enteropathogenic *E. coli* may cause food poisoning, especially in children under two years old [15].

According to the Bathing Water Directive (2006/7/EC) (BWD) [16], *E. coli* concentration in bathing water exceeding 500 CFU·100 mL^−1^ poses a high risk for bathers’ health. Concentration of *E. coli* > 250 and < 500 CFU·100 mL^−1^ poses medium hazard and concentration of *E. coli* < 250 CFU·100 mL^−1^ poses a low hazard.

In accordance with European and Polish national legal requirements [16], bathing water quality must be monitored. Reports published annually by the European Environment Agency (EEA) indicate that water quality in European bathing waters is high [17]; however, the situation in Poland is not so good. Up to 2010, coastal and transitional waters were often classified as posing a high risk for bathers’ health (*E. coli* concentration in bathing water exceeding 500 CFU·100 mL^−1^).

Microbiological monitoring of water quality is an essential tool for water quality assessment and health risk management. However, in its present form, it is time-consuming (sampling, inoculation, incubation, result readings), costly, and the results are obtained with a significant delay (more than 48 h). Unfortunately, these aspects are causing complications in managing bathing sites and maintaining the sanitary safety of bathers [18].

A practical model to forecast the cases of exceeding the norms for bathing waters on the basis of monitoring the data was presented by Džal et al. 2021 [19]. Authors underline that “the main problem with this modeling approach is to record a sufficient number of cases where bathing water quality is poor (exceedances) so that the data are more balanced”.

Another approach is the mathematical modeling of the spread of *E. coli* in the aquatic environment. The use of mathematical models describing the spread of pollutants in the environment has assisted environmental management for years. Modeling is a mathematical description of a phenomenon along with the determination of the spatial distribution of pollutant concentrations in the studied area (on a small scale, e.g., river section or pollutant discharge area, or on a global scale—the continent or the whole globe). The preparation of the model is a very complex and difficult issue, including both knowledge from the area of basic sciences (description of phenomena such as adsorption, desorption, diffusion, as well as information on chemical properties of compounds, survival of microorganisms in the environment, etc.) and interdisciplinary areas (knowledge of movement of air masses in the atmosphere, movement of water in rivers or sea basins, the condition of soil/sediment). The reliability of the obtained results depends mainly on the accuracy of the model used, its adaptation to the expected results, consideration of spatial and temporal dimensionality, and the amount and quality of the input data used for modeling. Models for the spread of microorganisms in different waterways (rivers, lakes, seas, and oceans), which have been used over the last 10 years, are presented in supplementary material, Section S1, Table S1 [20,21,22,23,24,25,26,27,28,29,30,31,32,33,34,35].

Currently, there is no standardized, universally applicable microbial pollution distribution model for marine coastal waters. An overview of the different models used for this purpose can be found in the work of Weiskerger et al. (2020) [36]; however, most of them were applied to freshwater (rivers and lakes), and only a few to estuaries and coastal waters. Therefore, the aims of this publication are:

1. To present results of the application of the PM3Dhydrodynamic model with the use of the microbiological module to predict the level of *E. coli* concentrations around the point of discharge of treated wastewater into the waters of the Gulf of Gdańsk, Poland;
2. To describe problems/barriers/obstacles encountered by researchers who undertake the development of a mathematical model of pathogen spread in the marine environment under conditions of discharging treated wastewater into brackish/saline water on the example of the Gulf of Gdańsk, Poland.

## 2. Results

*E. coli* is widely used as a fecal indicator organism. Their spread and survival in the environment depend on many factors such as temperature, solar insolation, hydrologic conditions, water chemistry, nutrient conditions, suspended and settled solids, and land-use practices [37].

The concentration of *E. coli* is important from a human health perspective. The Bathing Water Directive (2006/7/EC) (BWD) underlines that *E. coli* concentration in bathing water exceeding 500 CFU·100 mL^−1^ poses a high risk for bathers’ health.

Nag et al. (2021) indicate that a single exposure exceeding 500 CFU·100 mL^−1^ has a 10% chance of resulting in a gastrointestinal illness [38].

Given the widespread occurrence of fecal water contamination, with more information also coming from mathematical models, the implementation of effective management strategies for health should be possible and widely applied.

**Figure 1:**
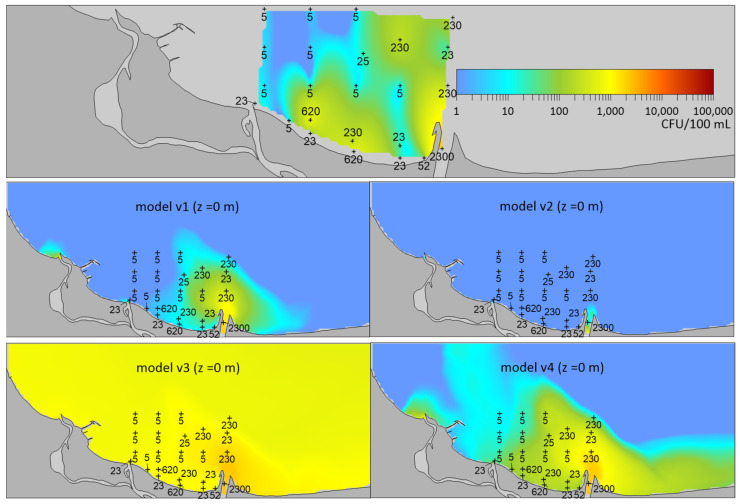
Comparison of *E. coli* concentrations observed on 29 May 2003 (**top** panel) and modeled with the use of different formulas for bacterial survival (versions: v1, v2, v3, v4, see Table 3).

The exemplary spatial distribution of *E. coli* calculated in the study area using four proposed versions of the model (Figure 1) shows the high influence of the chosen parametrization method on bacterial survival. The different versions used formulas describing *E. coli* survival rates previously used in different models (see Table 3). The comparison of the simulation results to the distribution of *E. coli* concentrations observed at the end of May 2003 points to the survival rate in the v2 version as being far too low, while in the v3 version too high. The final selection of the model version, i.e., the method of parameterization of bacterial survival in the model under development, was based on comparing the values of the modeled concentrations with the observed ones and a visual assessment of the consistency of spatial distributions made by several researchers.

Finally, the v4 [39] version of the model was selected as the most realistic description of the survival of *E. coli* bacteria.

### 2.1. Validation of the Model

Model validation for version v4 was performed by comparing model results with measurements of *E. coli* concentrations carried out in the Gulf of Gdansk from November 2002 to October 2003. Statistical accuracy characteristics such as bias, RMSE (root mean square error), and the correlation coefficient (r) were calculated for the decimal logarithm values of the measured and modeled concentrations at individual stations. (Table 1). The compatibility between model results and observations varied considerably on individual days. For example, the model underestimated the concentrations in November and December 2002 (BIAS = −1.94) while at the same time reporting the lowest statistical errors (RMSE). The correlation coefficient was very high in November (r = 0.96), while in December, it was negative (r = −0.46).

### 2.2. A Case Study Dated 12 August 2003—Spatial Distribution at Different Depths

**Figure 2:**
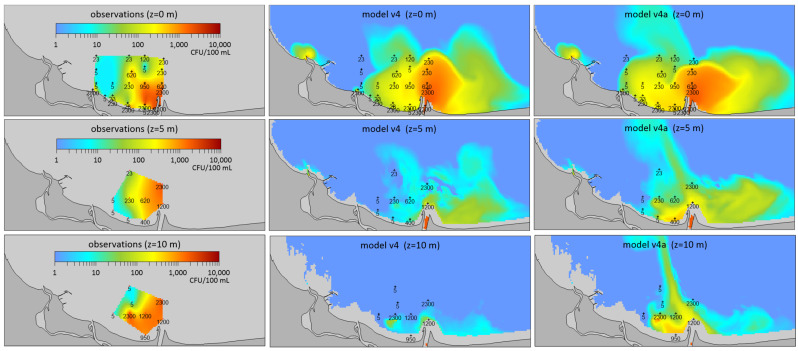
Distribution of *E. coli* concentration in the study area on 12 August 2003 at the depths of 0, 5, and 10 m; **left**: observed distributions; **center**: reference version of the model (v4); **right**: results of simulation modeled with assumed high concentration of *E. coli* in water flowing through the collector (v4a). Points indicate the location of the station and measured values.

During a series of tests on *E. coli* concentrations carried out at the outlet of the wastewater collector from the Gdańsk Wschód WWTP (Waste Water Treatment Plant), one case, dated 12 August 2003, differed significantly from the others. In particular, this applied to *E. coli* concentrations at depths of 5 and 10 m. In contrast to the measurements made on the other dates, the content of fecal bacteria was very high (up to 2300 CFU·100 mL^−1^) at that time (Figure 2).

The standard version of model calculations (v4) presented high concentrations in the foreland of the Vistula mouth only in the surface layer. However, measurements showed high concentrations of *E. coli* (up to 2300 CFU·100 mL^−1^) also at depths of 5 and 10 m. As fresh water in the Vistula spreads in a thin surface layer, it cannot be the source of high bacteria concentrations at these depths. The probable cause of such bacteria content may be the inflow of water from the WWTP via the collector. Measurements of *E. coli* concentrations performed on 12 August 2003 in wastewater discharged from the collector showed a concentration of 23,000 CFU·100 mL^−1^, and this value was used for model calculations. However, regarding these concentrations, only slightly higher values in the v4 model results were observed in the close vicinity of the collector outlet. It was hypothesized that bacterial content was significantly higher on the days before the study and assumed that in the period from 8 to 11 August, the concentrations were around 1,000,000 CFU·100 mL^−1^. With these assumptions, the results of the model, which were marked as the v4a version (Figure 2), indicated higher *E. coli* concentrations in the area of the collector and the Vistula mouth than in the reference version (v4), although simulated concentrations were still lower than the measured values. Model simulations showed that water with high concentrations of bacteria entering through the collector spread at a depth of 5–10 m towards the mouth of the Vistula and was then discharged towards the open waters of the Gulf of Gdańnsk. Such a distribution is consistent with observations, which confirms that the cause of high bacteria concentrations in subsurface waters could have been the inflow of large loads of sanitary pollutants on the days preceding the study.

## 3. Discussion

This research focuses on the problems and limitations that can be encountered in the process of preparing a mathematical model of the spread of biological contamination by *E. coli* bacteria in coastal seawater which can be an appealing alternative to expensive and time-consuming monitoring.

In our study, the main difficulty in creating a model of the spread of sanitary contamination, which was revealed by the analysis carried out in the study area, is the lack of the following data:

- *E. coli* concentration in river tributaries (including the largest one, the Vistula River);
- Results of systematic monitoring of *E. coli* concentration provided in treated wastewater discharged into the Gdańsk Bay.

As these concentrations can vary by an order of magnitude over very short periods of time, individual measurements may not be represented in the model validation database, and their absence can cause significant miscalculations in the model results. Multiplicity and variety in sources of inflow of the biological contamination additionally make the model more complex.

A mathematical model similar to ours was used by Locatelli et al. [20], who modeled the distribution of *E. coli* in coastal waters. In that study, bacteria entered the water with runoff from storm sewers. A constant concentration of the bacteria in the storm sewer (1 × 10^6^ CFU·100 mL^−1^) was assumed, many times higher than the concentration in coastal waters. The authors obtained better validation results (the correlation coefficient was 0.83 and RMSE: 0.66) compared to those obtained in the model presented in this paper (Table 3). However, it should be noted that modeling the inflow of contaminants with very high concentrations from a single source is much easier compared to modeling the inflow from multiple points and diffuse sources with relatively low concentrations. In the study conducted by Locatelli et al., modeling was carried out for only one point (Pont del Petroli) and only for the case of intense rainfall, which resulted in the inflow of large loads of *E. coli* through storm sewers [20]. A distinct source with very high loads of *E. coli* provides a more convenient case for modeling.

Another problem is the lack of data on loads of sanitary contamination from area sources, e.g., precipitation and feces of seagulls and other birds, as well as marine animals living in the study area (e.g., seals). Locatelli et al. indicated the presence of these bacteria in atmospheric air. Thus, heavy rainfall is expected to contribute significant loads of *E. coli* to the discharge area of the Gdańsk Wschód WWTP [20]. One of the problems inherent in prognostic models is the unpredictability of precipitation, i.e., the lack of knowledge about the time of occurrence of precipitation and its amount. In addition, stronger winds and rougher seas lead to shorter periods of seawater contamination [20,40], Locatelli et al. concluded that: “a great uncertainty is associated with the evaluated pollutant hazard, mainly due to the variability of water quality variables, rainfall patterns and seawater currents”.

The problems mentioned above indicate that the analysis of pathogen spread in seawater still implies a high degree of unpredictability, especially when several sources of contamination are considered in the model. 

**Figure 3:**
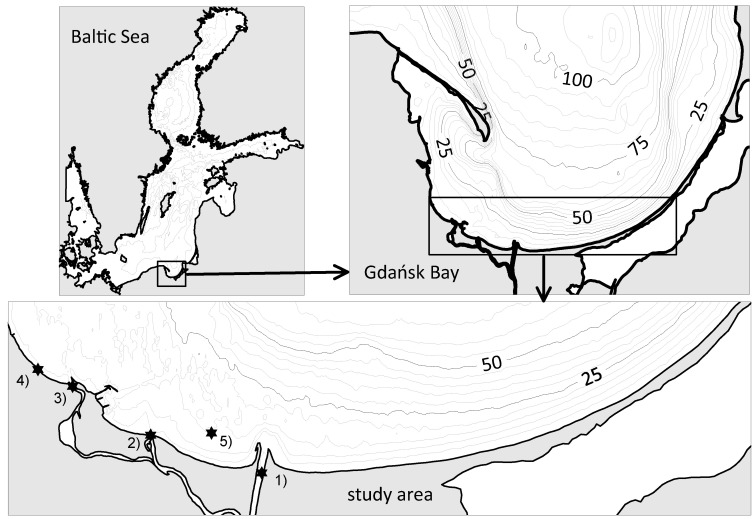
Bathymetry of the study area—the southern part of the Gulf of Gdańsk and areas modeled in lower resolution: the Baltic Sea and the Gulf of Gdańsk. Indicated locations are: (1) the mouth of the Vistula River, (2) the mouth of the Wisła Śmiała River, (3) the Martwa Wisła River, (4) the Jelitkowski Stream, and (5) the outlet from the collector of the Gdańsk Wschód WWTP.

The monitoring of the bathing waters shows a systematic decline in the level of *E. coli* concentration in the area of the study (Figure 3). Currently, *E. coli* concentrations higher than 250 CFU·100 mL^−1^ (low hazard for health) are observed very rarely. *E. coli* concentrations higher than 500 CFU·100 mL^−1^, with a high risk for bathers’ health, are found in case of accidents.

Nevertheless, efforts to develop such a model should be maintained in order to obtain a valuable tool for studying the spread of contaminants and identifying the most affected areas. The necessity of preparation and improvement of the mathematical model of the spread of microbiological contaminants (in particular *E. coli*) is justified by the occurrence of major accidents affecting the quality of the Baltic Sea water over the period of the last four years (Table 2).

In summer, the coastal beaches are used intensively for tourism (sunbathing, walking, and bathing). However, in the other seasons, the beaches are also frequently visited (weekly winter swimming, walking). Incidental discharges of untreated wastewater have forced the Sanitary Inspectorate to carry out intensive microbiological testing. Detection of high levels of pathogen contamination can lead to beach closures, with the resulting economic and public image losses for the region (especially in the summer season).

## 4. Materials and Methods

### 4.1. Description of the Study Area

The study area is situated in the southern part of the Gdańsk Bay, which forms part of the Gdańsk Basin located in the southern part of the Baltic Proper (Figure 3). The area is located between the mouth of the Vistula and the Wisła Śmiała and can be regarded as an estuary as its environmental conditions are influenced by both the brackish water of the Bay of Gdańsk (approx. 7 PSU) and the freshwater of the river. The water area affected by the Vistula is characterized by low salinity and is limited on the seaward side by a hydrological front, which restricts the spread of both the freshwater of the Vistula and the pollutants carried in this water [41]. The river’s fresh water, due to its lower density, flows on the surface over saline water, creating a clear vertical stratification of the water. The high variability of wind conditions results in a lack of constant surface currents in the Baltic Sea [42]. On short timescales, flows, especially surface ones, are determined by wind conditions. At longer scales, cyclonic systems of significant stability are observed. Their pattern is the result of depth distribution and freshwater inflow from land, and it can be noticed, for instance, in the Gdańsk Basin [43]. A more detailed description of hydrological conditions in the research area is provided in supplementary material, Section S4 [41,43,44,45,46,47].

### 4.2. Quality of the Bathing Water in the Gdańsk Bay Area

The microbiological quality of the water entering the Bay of Gdańsk is crucial both for bathing water quality and for the sanitary safety of thousands of people using beaches in that area. The water of the Gdańsk Bay is supplied from both point and area sources.

#### 4.2.1. Point Sources

The following point sources of *E. coli* inflow located in the Bay of Gdańsk are:

Four WWTPs (of which only two have a biological treatment stage);
the Vistula estuary and local water courses;
storm sewer.

#### 4.2.2. Area Sources

**Figure 4:**
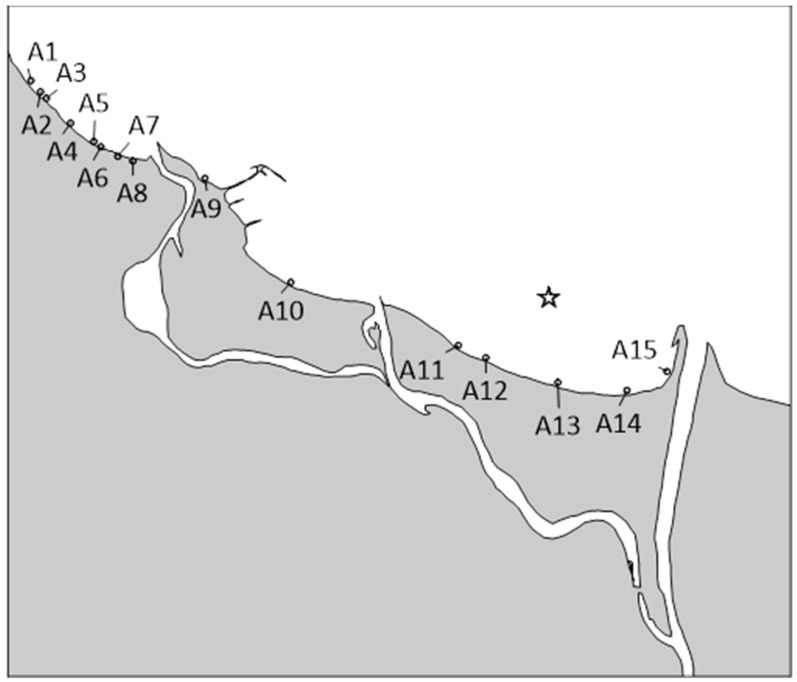
Location of bathing water sampling sites (A1–A15) with pipe outlet from the Gdańsk Wschód WWTP (star).

Area sources of microbiological inflow contamination are feces of birds (seagulls) and other animals (e.g., seals, dogs) and precipitation. There is currently no data on *E. coli* concentrations in rain; however, some information is available on *E. coli* content in the air at Gdańsk beaches in 2018. According to Michalska et al. [40], *E. coli* was detected in the range of 0–95 CFU·100 mL^−1^ the atmospheric air over sampling points A1–A15 (Figure 4).

It is estimated that the contribution of area sources to the inflow of *E. coli* into the Gdańsk Bay is negligible. In this case, the predominant sources of *E. coli* are the Vistula River and local water courses. The Gdańsk Wschód WWTP discharges less than 10% of a total load of microbiological contamination. Its collector was commissioned at the turn of 2002/2003, and since then, it has been covering beaches from A11 to A15. 

Bathing water monitoring used to be carried out by the Sanitary Inspectorate once a month between 2004 and 2020 at locations A1–A15 (Figure 2). Points A1–A8 are Tricity beaches located between Sopot and Gdańsk Brzeźno (a storm sewer is located at point A2), point A9 is Westerplatte beach, point A10 is Gdańsk Stogi beach, and points A11–A15 are beaches of Sobieszewo Island. 

**Figure 5:**
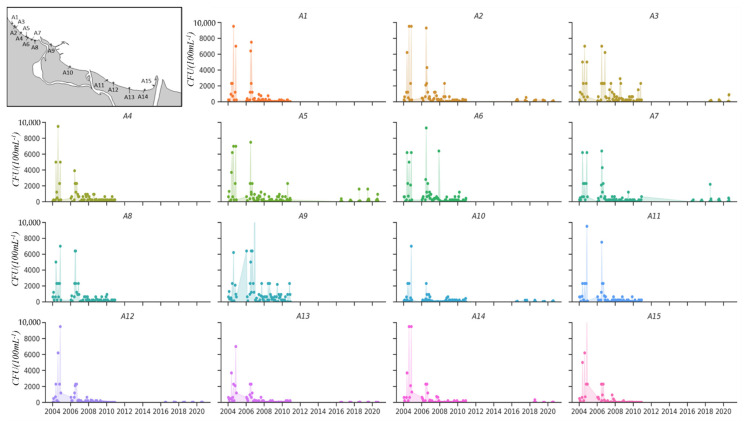
The average concentration of *E. coli* (CFU·100 mL^−1^) in bathing water of Gdańsk Bay for the period 2004–2020.

From 2004 to 2006, in the months from May to November, almost all beaches (A1 to A15) were contaminated with *E. coli* concentrations higher than 500 CFU·100 mL^−1^. According to the Bathing Water Directive (2006/7/EC) (BWD), such bathing water quality posed a high health risk (Figure 5). After 2007, *E. coli* concentrations at all locations decreased twofold. The occurrence of high levels of bacteria was sporadic and affected sites A2 and A3 (near the storm sewer) as well as A5 and A9.

The atmospheric deposition of *E. coli* by rain is difficult to estimate. The spatial distribution of annual precipitation in 2018 showed values ranging from 500 mm on Sobieszewo Island to 700 mm in the forested area of the moraine hills in the western part of Gdańsk [40]. Nevertheless, literature data show that *E. coli* concentration during a rainstorm can range from a few thousand to about twenty thousand CFU·100 mL^−1^ [48].

A more detailed description of the sources of *E. coli* inflow to the Gdańsk Bay is provided in supplementary material, Section S2 [49,50,51].

**Figure 6:**
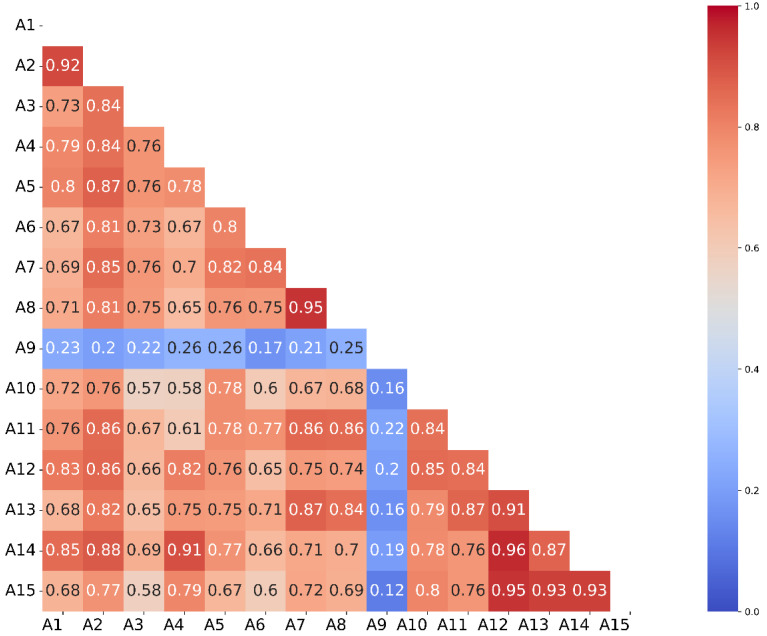
Correlation matrix for locations A1–A15.

Correlation analysis between *E. coli* concentration results in bathing water in the Gdańsk Bay (Figure 6).

Results from beach A9 are not correlated with the results of any other beach. Beach A10 shows a weak correlation with A11 and A12 and very weak with the other sites. A very strong correlation of results is observed for beaches A12–A15. This is the direct area of impact of the wastewater collector. A slightly weaker correlation of A11 results with points A12–15 suggests most likely the effect of wind directions dominating in this area (westerly and westerly-south winds). These winds displace the water at the surface and that below in an easterly direction, i.e., from A11 to A15.

## 5. Description of the Model for *E. coli* Spread

### 5.1. General Assumptions

The research methodology included the simulation of microbiological contaminant spread using the PM3D hydrodynamic model coupled with a microbiological module. This model simulated the spread of contaminants, especially *E. coli* concentrations, in relation to factors such as the volume of discharge from the Gdańsk Wschód WWTP, transport under the influence of sea currents, and turbulent diffusion. The mortality of bacteria in marine conditions, which depends on solar radiation input, temperature, and water salinity, was also taken into consideration.

The PM3D model [43] is a more recent version of the M3D model [52] developed by the Institute of Oceanography, University of Gdańsk. This model was derived from Blumberg and Mellor’s (1987) POM (Princeton Ocean Model) model and was also used as a basis for the ProDeMo (Production and Destruction of Organic Matter) eco-hydrodynamic model [53,54].

To simulate the sea current fields in the Gulf of Gdańsk, it is necessary to model the hydrodynamics of the entire Baltic Sea. However, modeling of the Vistula estuary and the collector area has to be done with a high spatial resolution (of the order of 100–200 m). Due to the time required for the calculations, the hydrodynamic conditions in the entire Baltic Sea cannot be modeled with such high resolution. Therefore, during this study, calculations were performed simultaneously for three domains, i.e., areas at different spatial resolutions (Figure 2). A previously developed model [28] with a resolution of 3 nautical miles (ca. 5.5 km) was used for calculations for the entire Baltic Sea. In the case of the Gulf of Gdańsk, a grid (domain) with a resolution of 0.5 nautical miles (ca. 0.9 km), previously used in upwelling studies, was adopted [55]. In contrast, a grid with a resolution of 0.1 nautical miles (ca. 185 m) was adopted to achieve high resolution in the Vistula estuary and the collector region.

In the computational domain of the southern part of the Gulf of Gdańsk, upstream flows from four streams (Vistula, Martwa Wisła, Wisła Śmiała, and Potok Jelitkowski) were taken into account (Figure 3). The flow values used for the Vistula were based on the daily measurements of the water level in Tczew. The average monthly flows were used in the case of the Martwa Wisła, Wisła Śmiała and Potok Jelitkowski. In the remaining computational domains, monthly average (1990–2000) inflows from about 200 of the largest rivers flowing into the Baltic Sea were taken into account. Zero salinity of river waters and climatic water temperature values were assumed.

The model takes into account the inflow of treated wastewater from the “Gdańsk Wschód” sewage treatment plant through the collector built in 2002–2003. This collector discharges treated water into the Gulf of Gdańsk. The distribution chamber is located at the position 18°52.232′ E, 54°22.29′ N. Two diffusers, each 218 m long, lead out of this chamber, placed horizontally above the bottom and perpendicular to each other. They are located approximately 3 m above the bottom at an approximate depth of 12 m, whereas the depth of the Bay is approximately 15 m. In the model, the position of the diffusers was taken into account in two different nodes of the computational grid for the southern part of the Gulf of Gdańsk. The survival rate of *E. coli* in the analyzed aquatic environment (Supplementary material, Section S3 [56,57,58,59,60,61,62,63]) is a key parameter in the mathematical model. The available literature reports that *E. coli* can survive in river water for up to 260 days in a wide range of temperatures (4–25 °C), while in brackish and saline waters, the survival rate decreases. The growth of the bacteria also depends on sunlight and the presence of organic matter [64].

The variation in the number of bacteria in a defined volume of water (N) over time is described by a function

*N* = *N*_0*e*_^−*kt*^

where *K* = the survival rate; *t* = time.

Various mathematical models use different empirical formulas to parameterize survival rates as a function of environmental conditions such as lighting, temperature, or water salinity.

The baseline mortality rate in the absence of light, used in those prognostic models, assumed a value in a wide range from 8.6 × 10^−5^ d^−1^ to 2.2 d^−1^ [20]. In this study, simulations were performed with four versions of the model (Table 3), adopting different formulas for the parameterization of the survival rate.

In order to select the optimal parameterization of mortality in the model, numerical simulations of the four versions of the model (designated as v1, v2, v3, and v4) were carried out for the study period related to the construction of the collector, i.e., from July 2002 to March 2003. According to Nowacki et al., the wastewater inflow from Gdańsk Wschód WWTP in 2002 reached 32,058.3 thousand m^3^·year^−1^ [68]. Due to the lack of specific data on the temporal variability of the WWTP inflow, a time-constant value of the water flow from the collector (1.01 m^3^·s^−1^) and in each diffuser tip (0.505 m^3^·s^−1^) was assumed for the calculations. Concentrations of fecal coliforms in wastewater discharged from the collector were calculated for each day on the basis of a linear interpolation of the values recorded on the days when measurements were taken Section S5 (Table S5). The simulations also included the inflow of *E. coli* into the Gulf of Gdańsk from the Vistula and the Wisła Śmiała River (Figure 2). The concentration of coliforms flowing into the Gulf of Gdańsk from these rivers was based on measurements made in their estuarial section (Section S5, Tables S3 and S4). In the periods between the measurements, the values resulting from linear interpolation of the measured bacterial concentrations were adopted for the simulation.

### 5.2. Faecal Coliforms (MPN of Faecal Coliforms)

The spatial distribution of fecal bacteria (ranges and directions of distribution) in the area under consideration was similar to that of coliforms, except that the size range was lower, ranging from <5 to 2300 (on average, the concentration difference between coliforms and fecal coliforms corresponds to one order of magnitude). The only exception is the results of tests carried out on 3 June 2003, when concentrations of fecal coliforms reached 23,000 CFU·100 mL^−1^ at points 9 and 25 (mouth of the Wisła Przekop). Fecal coliforms are commonly used in sanitary surveys as a basic indicator/marker of bacteriological water pollution. The value of this indicator/marker determines, in particular, the suitability of the bathing water. There are four popular bathing sites in the study area, the quality of which is systematically monitored by the Division of Environmental Toxicology, Medical University of Gdańsk.

## 6. Conclusions

This study focuses on the problems and limitations that can be encountered in the process of constructing a mathematical model describing the spread of biological contamination by *E. coli* bacteria in coastal seawater. The main difficulty in creating a model of the spread of sanitary contamination, which was revealed by the analyses carried out in the study area, is the lack of data regarding *E. coli* concentration in river tributaries (including the largest one, the Vistula River) and the lack of systematic monitoring of *E. coli* concentration in treated wastewater discharged into the Gdańsk Bay.

As these concentrations can vary by an order of magnitude over very short periods of time, individual measurements may not represent this adequately in the model validation database, and their absence can cause significant miscalculations in the model results. Additionally, multiple sources of biological contamination inflow make the model more complex.

Another problem is the lack of data on loads of sanitary contamination from area sources, e.g., precipitation and feces of seagulls and other birds, as well as marine animals living in the study area (e.g., seals). Locatelli et al. have pointed out the presence of this bacterium in atmospheric air. Thus, heavy rainfall is expected to contribute significant loads of *E. coli* to the discharge area of the Gdańsk Wschód WWTP [20]. 

One of the problems inherent in prognostic models is the unpredictability of precipitation, i.e., the lack of knowledge about the time of occurrence of precipitation and its amount. In addition, stronger winds and rougher seas lead to shorter periods of seawater contamination [20]. Locatelli et al. concluded that: “a great uncertainty is associated with the evaluated pollutant hazard, mainly due to the variability of water quality variables, rainfall patterns and seawater currents”.

## Figures and Tables

**Table 1:** Statistical compliance characteristics of the observed and modeled decimal logarithms of *E. coli* concentrations in the surface waters of the Gulf of Gdańsk in the period from November 2002 to October 2003.

DATE	BIAS	RMSE	R
5 November 2002	−1.94	0.28	0.96
10 December 2002	−1.94	0.26	−0.46
5 February 2003	−1.25	1.01	0.58
25 March 2003	−0.24	1.25	0.48
29 March 2003	−0.29	1.47	0.35
8 May 2003	0.08	1.20	0.17
10 May 2003	0.32	1.08	0.13
29 May 2003	0.18	0.86	0.37
17 June 2003	0.08	0.88	0.54
4 July 2003	−0.17	0.97	0.33
16 July 2003	0.33	1.00	0.19
12 August 2003	−0.63	1.18	0.29
6 September 2003	−0.14	1.17	0.26
26 September 2003	−0.61	1.25	0.22
3 October 2003	−0.41	0.98	0.23

**Table 2:** Failures of the wastewater network that have occurred in the last four years during the late spring and summer seasons.

Date	Area of Accident	Description
15 May 2018	Gdańsk Ołowianka wastewater pumping station	Engine failure at the pumping station managing 60% of the wastewater from Gdańsk and transporting it to the Gdańsk Wschód (WWTP). At that time, about 2600 m^3^ of wastewater was discharged per hour directly into Gdańsk Bay.
12 June 2019	outlet from Swarzewo WWTP	Pipe bursting and wastewater spilling into Puck Bay and onto residents’ properties
27 August 2019	the Vistula River	First pipeline failure in Warsaw
29 August 2020	the Vistula River	Second pipeline failure in Warsaw

**Table 3:** Mathematical formulas for the survival rate of *E. coli* used in different versions of the selected model.

Version of Model	Formula	Reference
v1	*k* = 6.466 − 0.195 *T +* 2.215·10^−7^ *T*^2^ *I*^2^	[65]
v2	$k=\frac{-\ln\left(0.1\right)}{31.305\cdot \exp\left(-0.17I_{UVA}\right)}$	[66]
v3	$k=\left(8.6\;\times \;10^{-5}\;+\;0.0026\;I\right)1.07^{T-20}$	[67]
v4	$k=\left(0.8+0.11\;S+0.0086\;I\right)1.07^{T-20}$	[39]

Where: *T*: water temperature; *I*, *IUVA*: total irradiance; *UVA*: irradiance; *S*: water salinity.

## Data Availability

Data contained within the article and supplementary materials are available on request from the authors.

## Supplementary Materials

The following are available online at https://www.mdpi.com/article/10.3390/molecules27144353/s1, Section S1. Table S1. Overview of mathematical models predicting water quality (including bathing water quality) available since 2010. Section S2. Area and point sources of *E. coli* in the research area Table S2. Characteristics of the treatment plant; Section S3. Bacterial survival in water, Section S4. Characteristics of hydrological conditions in the research area, Section S5. Description of the data used to create and validate the model; Table S3. The concentration of fecal coliforms (coli index) measured at the mouth Vistula; Table S4. ConcentrationS of fecal coliforms (coliform index) measured at the mouth Wisła Śmiała; Table S5. Concentrations of fecal coliform bacteria in treated sewage leaving the treatment plant as well as the results for the same bacteria at the sewage discharge point.

## Sample Availability

Not available.
