# Supplementary material for: Difficulties in the Modeling of E. coli Spreading from Various Sources in a Coastal Marine Area
Source: Molecules. 2022 Jul 7;27(14):4353. doi: 10.3390/molecules27144353 (PMC9316465; doi:10.3390/molecules27144353)
Supplement: Supplementary file 1 [file molecules-27-04353-s001.zip › molecules-1740813-SI.pdf]

molecules

MDPI

Article

Difficulties in Modelling of *E. coli* Spreading from Various Sources in the Coastal Marine Area

Lidia Wolska [1], Marek Kowalewski [2], Marta Potrykus [1], Bartosz Rybak [1,*] and Vlad Redko [1]

[1] Department of Environmental Toxicology, Faculty of Health Sciences, Medical University of Gdańsk, Dębowa Str. 23A, 80-204 Gdańsk, Poland; lidia.wolska@gumed.edu.pl (LW); bartosz.rybak@gumed.edu.pl (BR), marta.potrykus@gumed.edu.pl (MP), vlad.redko@gumed.edu.pl (VR);
[2] Institute of Oceanography, University of Gdańsk, Av. Marszałka Piłsudskiego 46, 81 - 378 Gdynia Poland, marek.kowalewski@ug.edu.pl (MK)
* Correspondence: bartosz.rybak@gumed.edu.pl; Tel.: +48 58 3491935

Citation: Wolska, L.; Kowalewski, M.; Potrykus, M.; Redko, V.; Rybak, B. Difficulties in Modelling of *E. coli* Spreading from Various Sources in the Coastal Marine Area. *Molecules* **2022**, *27*, 4353. https://doi.org/10.3390/molecules27144353

Academic Editors: Łukasz Chrzanowski and M. Gilles Mailhot

Received: 9 May 2022
Accepted: 1 July 2022
Published: 7 July 2022

Publisher's Note: MDPI stays neutral with regard to jurisdictional claims in published maps and institutional affiliations.

Copyright: © 2022 by the authors. Licensee MDPI, Basel, Switzerland. This article is an open access article distributed under the terms and conditions of the Creative Commons Attribution (CC BY) license (http://creativecommons.org/licenses/by/4.0/).

Supplementary Material

Section S1.

Table S1. Overview of mathematical models predicting water quality (including bathing water quality) available since 2010.

Model(s)	Year	Water type	Topic	Brief description	Reference
Modernized version MIKE URBAN model, compiled with InfoWorks ICM 8.5.	2020	Bathing waters affected by combined sewer overflows.	Modeling of *E. coli* distribution for hazard assessment of bathing waters affected by combined sewer overflows.	A coupled urban drainage and seawater quality model was developed, calibrated and validated based on local observations.	[1]
Bayesian Belief Network model	2020	Drinking water	A Bayesian Belief Network model to link sanitary inspection data to drinking water quality in a medium resource setting in rural Indonesia	The BBN model showed that the effect of holistic—combined interventions to improve the water quality were larger compared to individual intervention. The water quality at the POU was strongly related to the water quality at the POC and the effect of household water treatment to improve the water quality was more prominent in the context of better sanitation and hygiene conditions.	[2]
The environmental fluid dynamic code (EFDC), which was developed at the Virginia Institute of Marine Science	2020	Pond Water	Accounting for the Three-Dimensional Distribution of *Escherichia coli* Concentrations in Pond Water in Simulations of the Microbial Quality of Water Withdrawn for Irrigation	This model has been successfully applied to a wide range of environmental studies simulating the variation of hydrodynamic and water quality in lakes, rivers, estuaries, reservoirs, ponds and wetlands.	[3]
Delft3D-WAQ	2020	River deltas	Numerical investigation of the spatial distribution of *Escherichia coli* in river deltas for different values of river discharge, temperature and irradiation of the water surface	Overall, full models built using ensemble (e.g., Node Harvest) and "black-box" (e.g., SVMs) learners out-performed full models built using more interpretable learners (e.g., tree- and rule-based learners) for both outcomes. However, nested *eaeA-stx* models built using interpretable learners and microbial data performed almost	[4]

Node Harvest, SVMs	2020	North-eastern Streams Used to Provide Water for Produce Production	Predictive Models May Complement or Provide an Alternative to Existing Strategies for Assessing the Enteric Pathogen Contamination Status of North-eastern Streams Used to Provide Water for Produce Production as well as these full models.	The hydrodynamic model Delft3D and the water quality model Delft3D-WAQ were used for the investigation. Delft3D solves the unsteady shallow water equations in two (depth averaged) dimensions.	[5]
Bayesian multiple linear regression	2019	River	Prediction Of Fecal Indicator Organism Concentrations In Rivers: The Shifting Role Of Environmental Factors Under Varying Flow Conditions.	Bayesian multiple linear regression approach that complies with the European Bathing Water Directive	[6]
MIKE 21 FM-ECOLab	2018	River	Modelling of river faecal indicator bacteria dynamics as a basis for faecal contamination reduction	MIKE 21 FM-ECOLab model was applied to understand river indicator bacteria (*E. coli* and enterococci) dynamics. The model compares very well to bacteria measurements from the study area.	[7]
The Hydrological Simulation Program – FORTRAN	2017	River	Integrated hydro-bacterial modelling for predicting bathing water quality	The model has been compiled from 5 sub-models: HSPF, Info works model, DMHSF model, One-dimensional River	[8]
STELLA 9.1.3, the dynamic model of the PDAM distribution network	2017	Water distribution network	Dynamic modelling of *E. coli* growth in water distribution system (a case study: water supply zone 3 in Surabaya Indonesia	Modeling applying in *E. coli* growth prognosis in water distribution system (water supply zone) in Surabaya, Indonesia.	[3]
SWAT and Watershed Assessment Model (WAM)	2016	Surface waters	Modeling fate and transport of fecally-derived microorganisms at the watershed scale: State of the science and future opportunities	Developments in the microbial water quality modelling over last 10 years are reviewed	[9]
General process-based watershed-scale solute transport modeling.	2017	Surface waters	Modeling watershed-scale solute transport using an integrated, process-based hydrologic model with	Distributed hydrologic models that simulate fate and transport processes at sub-daily timescales are useful tools for estimating pollutant loads exported from watersheds to lakes and oceans downstream. There has	[10]

			applications to bacterial fate and transport	been considerable interest in the application of integrated process-based hydrologic models in recent years.	
Own mathematically described model regards *E. coli* migration	2015	*Water environment*	Modelling the mechanics and hydrodynamics of swimming *E. coli.*	The investigation towards to mechanisms of *E. coli* behavior in water environment.	[7]
SLIM-EC2	2014	Rivers	Integrated modelling of faecal contamination in a densely populated river–sea continuum (Scheldt River and Estuary)	The resulting model (SLIM-EC2) presents two specific and new features compared to the older SLIM-EC model version. The first is that the *E. coli* concentrations in the river are split in three fractions: the free *E. coli* in the water column, the ones attached to suspended solids and those present in the bottom sediments, each with their own transport, decay and settling-resuspension dynamics.	[11]
Recently developed Barrage module (EFDC_B)	2014	The Bristol Channel and Severn Estuary	Refinements To The Efdc Model For Predicting The Hydro-Environmental Impacts Of A Barrage Across The Severn Estuary.	Details are given of a barrage module being implemented into the EFDC model to represent the various hydraulic structures, such as turbines and sluice gates, as deployed along the barrage line.	[12]
New marine water quality forecasting system for real-time and short-term predictions	2014	Marine water	An integrated catchment-coastal modelling system for real-time water quality forecasts	The integrated model is validated in an Irish catchment-coastal system using hydrodynamic and water quality data. The forecasting system was then used to provide short-term and real-time forecasts of *E. coli* and Intestinal Enterococci concentrations (IE) in the near-shore coastal waters of Bray, Ireland. Two hindcast scenarios were simulated: 5F in which predictions were based on rainfall forecasts only; and I-5F where forecasts of 5F were improved by incorporating real-time data. Results indicate that predictions of *E. coli* of scenario I-5F are improved.	[13]
SENEQUE-EC	2013	Rivers	Modelling Faecal Contamination In The Scheldt Drainage Network	The microbiological module describes the sources of E. coli, their transport and the processes responsible for the fate of *E. coli* once released into the natural environment (mortality, settling and resuspension). This	[11]

				model differentiates the dynamics of three types of *E. coli*: free-floating *E. coli, E. coli* attached to suspended solids in the water column and *E. coli* present in sediments.	
One-dimensional model in combination with the advection – dispersion equation	2013	Natural waters	Modeling Transport Of *Escherichia coli* In A Creek During And After Artificial High-Flow Events: Three-Year Study And Analysis	A conservative tracer difluoro benzoic acid (DFBA) was added to the released water in 2009 and 2010. Water flow rate, *E. coli* and DFBA concentrations as well as water turbidity were monitored with automated samplers at three in-stream weirs. A one-dimensional model was applied to simulate water flow, and E. coli and DFBA transport during these experiments. The Saint-Venant equations were used to calculate water depth and discharge while a stream solute transport model accounted for release of bacteria by shear stress from bottom sediments, advection-dispersion, and exchange with transient storage (TS).	[14]
3D Deterministic Model	2013	Beach water	Real-time forecasting of Hong Kong beach water quality by 3D deterministic model	A 3D deterministic hydrodynamic model is developed to provide daily water quality forecasting for eight marine beaches in Tsuen Wan, which are only about 8 km from the Harbour Area Treatment Scheme (HATS) outfall discharging 1.4 million m3/d of partially-treated sewage.	[15]
SWAT	2012	River	PhD thesis:<br>Modeling In- Stream *Escherichia coli* Concentrations	Formulations were programmed in FORTRAN language, and were integrated into the Soil and Water Assessment Tool (SWAT)	[16]

Section S2.

Area and point sources of *E.coli* in the research area

In order to analyze the solid pollutants in the Gulf of Gdańsk, potential point and area sources of *E. coli* influx were analyzed.

Point sources

Sewage treatment plants

Sewage from four treatment plants is discharged into the waters of the Bay of Gdańsk. Basic parameters characterizing these sewage treatment plants are given in Table S2.

Table S2. Characteristics of the treatment plant (source: Ratajczyk W., 201717)

Parameter	Sewage treatment plants			
	Gdańsk Wschód	Dębogórze	w Jastarni	w Helu
year of creation	1976	1964	1992	2000
type	mechanical-biological with chemical support of phosphorus removal	mechanical-biological with chemical support of phosphorus removal	mechanical-biological with chemical support of phosphorus removal	mechanical-biological with chemical support of phosphorus removal
Wastewater discharged to the bay [m3/year]	35 040 000	20 077 000	445 252	289 000
Population in the catchment area	500 000	360 000	4 000*	3 800*
Discharge of the outlet from the treatment plant	2.5 km<br>12 m depth	2.5 km<br>8 m depth	1.26 km<br>6 m depth	30 m<br>1 m depth

* in the summer season, the number of people using the sewage network increases even > 10 times

The Vistula River and other watercourses

The inflow of waters - characteristics, the Vistula and other watercourses

Vistula and 10 other rivers and canals with storm water from the city of Gdańsk and the surrounding towns flow directly into the waters of the Gdańsk Bay. The river basin of the Vistula comprises almost the whole eastern part of Poland. River length amounts to 1047 km from its source to the discharge point. The whole basin has an area 194 thousand km^2, and is inhabited by 24.5 million people. The average annual discharge of the Vistula to the Bay of Gdansk is more than a thousand cubic meters per second [18].

Area sources

Animals (birds, seals, dogs and more)

The daily number of water birds living in the Bay of Gdańsk (according to the KULING Water Bird Research Group, Poland) ranges from several to several dozen thousand individuals per day, depending on the season, leg, flights and weather conditions [19].

It is difficult to estimate how much fecal excretion by birds over the waters of the gulf contributes as a source of *E. coli* strains. The microflora of the digestive tract of water birds depends on their type of diet. The digestive tract flora of individual groups of birds will depend on the morphological and ecological groups of birds. *E. coli* strains can be found

in the diet of bentophages, phytophages and ichthyophages. Moreover, omniphages, whose diet contains products of human origin, e.g. from municipal waste, may likewise be a certain source of *E. coli*. Among bentophages or phytophages, the titer of *E. coli* excreted will vary depending on the feeding site. In conclusion, in our opinion, area sources do not constitute a significant influx of *E. coli* to the waters of the Bay of Gdańsk.

Precipitation

The amount of *E.coli* in rainfall in the Gdańsk Bay area was assessed on the basis of microbiological studies of aerosol by Michalska et al. 2018 [20] and was not significant for the development of the model.

Section S3.

Bacterial survival in water

Species of *E. coli* are able to survive in river water for up to 260 days over a wide range of temperatures (4-25°C), and significantly, the lower the temperature, the higher the survival rate of the bacteria [21]. In addition, these bacteria are able to survive in river sediments. According to studies, river sediments are a rich nutrient source for them, but the pH of the sediment is crucial for the length of their survival [22]. The survival of *E. coli* in water and/or river sediments is also affected by other factors such as the occurrence of predatory organisms, the presence of other bacteria inhabiting the same ecological niche as well as the amount of organic compounds available to the microorganisms [23]. High levels of organic matter can stimulate the growth of *E. coli* bacteria disproportionately. It has also been proven that *E. coli* bacteria attach to particles of suspended matter in river water, and the amount of matter in river water correlates with an increased presence of *E. coli* in this fraction [24]. Research has shown that the rate of bacteria sinking with suspended matter was relatively constant at higher concentrations of suspended matter and equalled 0.066 $m \cdot h^{-1}$. Additionally, it was noted that the survival rate of *E. coli* was two times higher if the bacteria were bound to suspended matter particles compared to free-living bacteria [24]. Sagarduy et al. (2019) carried out studies on the survival of *E. coli* in river and seawater, which showed that, depending on sampling location, the survival (T90) of these bacteria ranged from 3 to 69 h. Researchers pointed out that solar radiation had the greatest influence on the survival rate, so this parameter was introduced into the model for assessing the survival of *E. coli* in water basins. This study also highlighted the fact that bacterial cells went into a state of "numbness" (they were viable but could not be cultured), making it difficult to truly assess the number of live cells in a sample [25]. On the other hand, Sagarduy et al. also indicated many factors affecting the survival of *E. coli* in marine and surface water such as temperature, solar radiation, water chemistry, dissolved matter, hydrological conditions, nutrient availability and land management around the reservoir [25]. In marine water, solar and UV radiation are thought to be the most significant factors affecting bacterial survival [26]. Two reference *E. coli* strains tested showed much greater sensitivity to the presence of radiation than to changes in temperature or salinity in seawater samples, demonstrating a reduction in the survival of these strains by 15-70 times compared to results of experiments conducted in darkness. In contrast, Korajkic et al. showed that the microbiota inhabiting a given water body had a significant impact on the rate of extinction of allochthonous *E. coli*. This work also confirmed that the die-off of *E. coli* in freshwater progressed more slowly than in saltwater, but pointed out that the presence of aquatic microbiota significantly affected the survival rate of these indicator bacteria by lowering it [27]. Lothigius et al. investigated the survival of clinical *E. coli* isolates in fresh and salt water in Sweden. The survival of 6 *E. coli* ETEC isolates was significantly higher in freshwater than in seawater during the 3-month study. The number of bacteria decreased in both cases, but in freshwater the value dropped from 109 to 106 CFU mL^{-1} whereas in salt water it was reduced to 101 CFU mL^{-1} [28].

Section S4.

Characteristics of hydrological conditions in the research area

The study area is situated in the southern part of the Gdańsk Bay, which forms part of the Gdańsk Basin located in the southern part of the Baltic Proper. The area is located between the mouth of the Vistula and the Wisła Śmiała and can be regarded as an estuary as its environmental conditions are influenced by both the brackish water of the Bay of Gdańsk (approx. 7 PSU) and the fresh river water. The water area affected by the Vistula is characterized by low salinity and is limited on the seaward side by a hydrological front, which restricts the spread of both fresh Vistula water and pollutants carried in this water [29]. Fresh river water, due to lower density, flows on the surface over saline water, creating a clear vertical stratification of the water. The observed vertical stratification significantly impedes the mixing processes, what greatly affects the dispersion of wastewater discharged through the collector.

The spread of microbiological contaminants in the environment of the Gdańsk Bay may also be affected by the prevailing wind directions and associated sea currents. The location of the Baltic Sea in a temperate climatic zone results in a predominance of western and south-western winds. While analysing the prevailing wind directions, Taranowska et al. distinguished three periods during the year [30]:

- spring, when there is a large variation in circulation directions, with easterly and north-easterly winds being the most prevalent ones;
- summer, when westerly winds blow most frequently, but northerly winds are also frequent;
- autumn and winter, when south-westerly winds are more common [39].

On a yearly basis, cyclonic circulation dominates, causing the predominance of westerly and south-westerly winds [31,32].

Wind speeds blowing over the Baltic Sea show high spatial and seasonal variability [33]. Two seasons can be distinguished during the year: (i) late autumn and winter, when frequent storms occur, causing the average speed in the high seas zone to reach 9 $m^{-1}\cdot s$, and (ii) spring-summer, when winds are much weaker (approx. 5 $m^{-1}\cdot s$) and storms appear sporadically.

On short timescales, flows, especially surface ones, are determined by wind conditions. At longer scales, cyclonic systems of significant stability are observed. Their pattern is the result of depth distribution and freshwater inflow from land and it can be noticed, for instance in the Gdańsk Basin [34].

Section S5

Description of the data used to create and validate the model

The data used for the model were:

-results published in literature;

-a project commissioned by Saur Neptun Gdańsk from the Institute of Marine and Tropical Medicine in 1999;

-results of bathing water tests carried out by the Voivodeship Sanitary and Epidemiological Station in Gdańsk;

Tables S3-1, S3-2 present the concentration of faecal coliforms and coli index in two main river tributaries to Zatoka Gdańska and table S3-3 presents concentrations of faecal coliform bacteria in treated sewage leaving the treatment plant as well as the results of the determination of the same bacteria at the sewage discharge point.

Table S3. Concentration of faecal coliforms (coli index) measured at the mouth of Vistula

Date	coliform index at 100mL	coli index
16-07-2002	9500	2300
05-11-2002	23000	23000
10-12-2002	2300	23
05-02-2003	620	230
25-03-2003	95000	62000
29-03-2003	9500	9500

Table S4. Concentration of faecal coliforms (coliform index) measured at the mouth of Wisła Śmiała

Date	coliform index at 100mL	coli index
16-07-2002	230	230
05-11-2002	23000	2300
10-12-2002	23	23
05-02-2003	2300	230
25-03-2003	2300	23
29-03-2003	23	<5

Table. S5- Concentrations of faecal coliform bacteria in treated sewage leaving the treatment plant as well as the results for the same bacteria at the sewage discharge point

Date	coliform index at 100mL treated sewage leaving	coli index treated sewage leaving
05-11-2002	230 000	230 000
10-12-2002	230 000	230 000
05-02-2003	2 300 000	2 300

25-03-2003	230 000	230 000
29-03-2003	23 000	23 000

Data	coli index at 100mL effluent from the treatment plant
16.07.2002	23 000
05.11.2002	230 000
10.12.2002	230 000
05.02.2003	2 300
25.03.2003	230 000
29.03.2003	60 000
08.05.2003	23 000
10.05.2003	50 000
29.05.2003	23 000
17.06.2003	23 000
04.07.2003	2 300 000
16.07.2003	23 000
12.08.2003	23 000

References

1. Locatelli, L.; Russo, B.; Acero Oliete, A.; Sánchez Catalán, J.C.; Martinez-Gomariz, E.; Martinez, M. Modeling of *E. coli* distribution for hazard assessment of bathing waters affected by combined sewer overflows. Nat. Hazards Earth Syst. Sci. 2020, 20, 1219–1232.
2. Daniel, D.; Iswarani, W.P.; Pande, S.; Rietveld, L. A Bayesian Belief Network model to link sanitary inspection data to drinking water quality in a medium resource setting in rural Indonesia. Sci. Rep. 2020, 10, 18867.
3. Srikandi, E.D.; Karnaningroem, N. Dynamic modelling of *Escherichia coli* growth in water distribution system (a case study: Water supply zone 3 in Surabaya Indonesia). Int. J. Adv. Res. 2017, 5, 1145–1149.
4. Pandey, P. Modeling in- stream *Escherichia coli* concentrations. Diss. Abstr. Int. 2012, 74, 5. suppl. B:1–336.
5. Stocker, M.D.; Jeon, D.J.; Sokolova, E.; Lee, H.; Kim, M.S.; Pachepsky, Y.A. Accounting for the Three-Dimensional Distribution of *Escherichia coli* Concentrations in Pond Water in Simulations of the Microbial Quality of Water Withdrawn for Irrigation. Water 2020, 12, 1708.
6. Herrig, I.; Seis, W.; Fischer, H.; Regnery, J.; Manz, W.; Reifferscheid, G.; Böer, S. Prediction of fecal indicator organism concentrations in rivers: The shifting role of environmental factors under varying flow conditions. Environ. Sci. Eur. 2019, 31, 59.
7. Hu, J.; Yang, M.; Gompper, G.; Winkler, R.G. Modelling the mechanics and hydrodynamics of swimming *E. coli*. Soft Matter 2015, 11, 7867–7876.
8. Huang, G.; Falconer, R.A.; Lin, B. Integrated hydro-bacterial modelling for predicting bathing water quality. Estuar. Coast. Shelf Sci. 2017, 188, 145–155.
9. Cho, K.H.; Pachepsky, Y.A.; Oliver, D.M.; Muirhead, R.W.; Park, Y.; Quilliam, R.S.; Shelton, D.R. Modeling fate and transport of fecally-derived microorganisms at the watershed scale: State of the science and future opportunities. Water Res. 2016, 100, 38–56.
10. Niu, J.; Phanikumar, M. Modeling watershed-scale solute transport using an integrated, process-based hydrologic model with applications to bacterial fate and transport. J. Hydrol. 2015, 529, 35–48.
11. De Brauwere, A.; Gourgue, O.; de Brye, B.; Servais, P.; Ouattara, N.K.; Deleersnijder, E. Integrated modelling of faecal contamination in a densely populated river-sea continuum (Scheldt River and Estuary). Sci. Total Environ. 2014, 468–469, 31–45.
12. Zhou, J.; Falconer, R.A.; Lin, B. Refinements To The Efdc Model For Predicting The Hydro-Environmental Impacts Of A Barrage Across The Severn Estuary. Renew. Energy 2014, 1, 458–476.
13. Bedri, Z.; Corkery, A.; O'Sullivan, J.J.; Alvarez, M.X.; Erichsen, A.C.; Deering, L.A.; Demeter, K.; O'Hare, G.M.P.; Meijer, W.G.; Masterson, B. An Integrated Catchment-Coastal Modelling System for Real-Time Water Quality Forecasts. Environ. Model. Softw. 2014, 61, 458–476.

14. Yakirevich, A.; Pachepsky, Y.A.; Guber, A.K.; Gish, T.J.; Shelton, D.R.; Cho, K.H. Modeling transport of *Escherichia coli* in a creek during and after artificial high-flow events: Three-year study and analysis. Water Res. 2013, 47, 2676–2688.
15. Chan, S.N.; Thoe, W.; Lee, J.H.W. Real-time forecasting of Hong Kong beach water quality by 3D deterministic model. Water Res. 2013, 47, 1631–1647.
16. Pandey, P.K.; Soupir, M.L.; Ikenberry, C.D.; Rehmann, C.R. Predicting Streambed Sediment and Water Column *Escherichia coli* Levels at Watershed Scale. JAWRA J. Am. Water Resour. Assoc. 2016, 52, 184–197.
17. Ratajczak, W. Environmental Threats Resulting from the Discharge of Treated Sewage to Sea Waters. Ph.D. Thesis, Medical University of Gdańsk, 2017. Unpublished. (In Polish).
18. Majewski, W. General characteristics of the Vistula and its basin. Acta Energetica 2013, 2, 6–15.
19. Meissner, W.; Kośmicki, A.; Bzoma, S. Liczebność ptaków wodnych na Zatoce Gdańskiej w okresie wrzesień 2020–kwiecień 2021. Ornis Pol. 2021, 62, 357–363.
20. Flint, K.P. The long-term survival of *Escherichia coli* in river water. J. Appl. Bacteriol. 1987, 63, 261–270.
21. Michalska, M.; Zorena, K.; Bartoszewicz, M. Analysis of faecal bacteria isolated from air and seawater samples following an emergency sewage discharge into the Gulf of Gdansk in 2018-preliminary study. *Int. Marit. Health* **2019**, *70*, 239–243.
22. Liang, C.; Yao, Z.; Du, S.; Hong, M.; Wang, K.; Zhang, D. Sediment pH, not the bacterial diversity, determines *Escherichia coli* O157:H7 survival in estuarine sediments. Environ. Pollut. 2019, 252, 1078–1086.
23. Wanjugi, P.; Fox, G.A.; Harwood, V.J. The Interplay Between Predation, Competition, and Nutrient Levels Influences the Survival of *Escherichia coli* in Aquatic Environments. Microb. Ecol. 2016, 72, 526–537.
24. Garcia-Armisen, T.; Servais, P. Partitioning and fate of particle-associated *E. coli* in river waters. Water Environ. Res. 2009, 81, 21–28.
25. Sagarduy, M.; Courtois, S.; Del Campo, A.; Garmendia, J.M.; Petrau, A. Differential decay and prediction of persistence of *Enterococcus* spp. and *Escherichia coli* culturable cells and molecular markers in freshwater and seawater environments. Int. J. Hyg. Environ. Health 2019, 222, 695–704.
26. Rozen, Y.; Belkin, S. Survival of enteric bacteria in seawater. FEMS Microbiol. Rev. 2001, 25, 513–529.
27. Korajkic, A.; Wanjugi, P.; Harwood, V.J. Indigenous microbiota and habitat influence *Escherichia coli* survival more than sunlight in simulated aquatic environments. Appl. Environ. Microbiol. 2013, 79, 5329–5337.
28. Lothigius, A.; Sjöling, A.; Svennerholm, A.-M.; Bölin, I. Survival and gene expression of enterotoxigenic Escherichia coli during long-term incubation in sea water and freshwater. J. Appl. Microbiol. 2010, 108, 1441–1449.
29. Matciak, M.; Nowicki, J. The hydrological and hydrochemical division of the surface observations. Oceanologia 1996, 37, 75–88.
30. Taranowska, S. O kierunkach i prędkościach wiatrów dolnych na obszarze Środkowego i Południowego Bałtyku (1947–1960). Przegląd Geogr. 1968, 13, 75–88.
31. Kullenberg, G. Physical Oceanography. In The Baltic Sea; Voipio, A., Ed.; Elsevier Oceanography Series; Elsevier: 1981; Volume 30, pp. 135–181.
32. Voipio, A. (Ed.) The Baltic Sea; Elsevier Oceanography Series; Elsevier: 1981; Volume 30.
33. Kwiecień, K. Warunki klimatyczne. In Bałtyk Południowy; Ossolineum: Wrocław, Poland, 1987; pp. 219–288.
34. Jędrasik, J.; Kowalewski, M. Mean annual and seasonal circulation patterns and long-term variability of currents in the Baltic Sea. J. Mar. Syst. 2019, 193, 1–26.
